# A Randomized, Double-Masked, Active-Controlled, Crossover Phase III Equivalence Study of Generic Dorzolamide 2% versus Innovator Trusopt® Eye Drop Solution in Subjects with Open-Angle Glaucoma or Ocular Hypertension

**DOI:** 10.1155/2022/5249922

**Published:** 2022-07-20

**Authors:** Katharina Bell, Christina Korb, Christina Butsch, Bert Constantin Giers, Anna Beck, Alicja Strzalkowska, Christian Ruckes, Ulrike Klingberg, Norbert Pfeiffer, Katrin Lorenz

**Affiliations:** ^1^Department of Ophthalmology, University Medical Center, Johannes Gutenberg-University Mainz, Mainz, Germany; ^2^Interdisciplinary Center Clinical Trials Mainz, University Medical Center, Johannes Gutenberg-University Mainz, Mainz, Germany; ^3^Alfred E. Tiefenbacher GmbH & Co. KG, Hamburg, Germany

## Abstract

**Background:**

The aim of this study was to demonstrate the equivalence of generic dorzolamide 2% eye drops solution versus the innovator formulation (Trusopt® eye drops solution) in patients with open-angle glaucoma or ocular hypertension.

**Methods:**

This prospective, monocentric, double-masked, active-controlled crossover phase III study included 32 patients. After washout, patients were randomized to reference product (Trusopt®) or test product (dorzolamide 2% eye drops, Rompharm Company SRL) for a 4-week period. Subsequent washout and crossover were performed. Drops were applied t.i.d. The primary efficacy endpoint was the difference in mean diurnal IOP. Goldmann applanation tonometry was performed at 8 am, 12 pm, and 4 pm at each visit, and safety was assessed by documentation of adverse events (AEs). Therapy adherence was documented by self-reporting and eye drop bottle weighing. An ANOVA with treatment, sequence, study period, and patient within the sequence as effects was performed and an additional post hoc ANCOVA including the baseline IOP was also performed.

**Results:**

34 patients were randomized and analyzed in the safety population. The per-protocol population included 32 patients. According to the self-report, all patients were >80% compliant. Under the ANCOVA model, the 90% confidence interval for the average change of the IOP −0.27 mmHg (−1.17 mmHg–0.64 mmHg) is included by the acceptance range −1.5 mmHg to +1.5 mmHg after excluding 2 patients, which had falsely reported high therapy adherence. No clinically relevant difference was observed in frequency or severity of the AEs between both treatments.

**Conclusions:**

This study showed the equivalence of the tested generic dorzolamide 2% eye drops solution to the reference product Trusopt® eye drops solution. *Trial Registration.* This trial is registered with (ClinicalTrials.gov (identifier: NCT00878917) on April 9, 2009).

## 1. Background

Glaucoma is a leading cause of blindness and represents a group of ocular disorders characterized by progressive loss of retinal ganglion cells and their axons. This optic nerve degeneration results in gradual loss of visual field, which may ultimately lead to blindness [1–3]. The pathogenesis of glaucoma is partially understood. Elevated intraocular pressure (IOP) is the most relevant risk factor for disease development or progression and at this moment in time, in addition, represents the only clinically addressable factor [4–10]. This also is the case for patients suffering from normal-tension glaucoma (NTG), which shows glaucomatous optic nerve damage despite normal IOP [11].

Pharmacological treatment with topical hypotensive drugs is generally considered to be the first-line therapy for newly diagnosed glaucoma patients. In patients not sufficiently controlled with monotherapy, drugs with different mechanisms of action are combined to reach an additive effect [12]. Depending on the individual disease severity and the rate of progression, an individualized target IOP is evaluated for each patient, aiming to reach not only this target IOP with the maximum amount of safety and efficacy but also including economic consideration [13]. Several classes of IOP-lowering topical agents are available and first-choice treatments include prostaglandins, beta-blockers, adrenergic antagonists, and carbonic anhydrase inhibitors (CAIs) [14–19]. The first topical CAI available was dorzolamide which was approved by the FDA (New Drug Application (NDA): 020408) in 1994 [14]; however, a dose-response study was performed several years beforehand [20]. It was shown to be safe and effective as a primary therapy as well as in combination with other IOP-lowering eye drops [21,22]. Concerning the cost efficiency of glaucoma treatment with topical eye drops, generic medications often are taken into consideration, as these can be distributed at a much lower cost than the innovator preparation [23]. Although cost efficiency is a major benefit of generic drugs, special care must be taken concerning equal efficacy and equal risk profile of the generic drug. Bioequivalence studies prove equivalence in terms of efficacy and safety. Such clinical studies are not only crucial for the registration of these drugs but also provide and improve confidence of health professionals and patients when making use of generic drugs.

The aim of this study was to demonstrate the equivalence of generic dorzolamide 2% eye drops solution versus the innovator formulation (Trusopt® eye drops solution) in subjects with open-angle glaucoma or ocular hypertension. According to the regulations of the European Medicines Agency (EMA), which assesses applications to market generic medicines in the European Union (EU), a generic medicine needs to contain the same active substance and the same dose, as the reference medicine (https://www.ema.europa.eu/en) [24]. Proof of equivalence of this generic formulation was mandatory for registration in a decentralized application in 11 European countries, as the viscosity of the generic eye drops is slightly lower compared to the innovator formulation.

## 2. Methods

This prospective, monocentric, double-masked, active-controlled crossover phase III study was performed at the Department of Ophthalmology, University Medical Center, Johannes Gutenberg University of Mainz, Germany. Scientific advice by the competent authorities in Germany was obtained before the finalization of the study protocol as the aim of this study was the registration of generic dorzolamide 2% eye drops solution in a decentralized application. Approvals by the Ethics Committee of “Landesärztekammer Rheinland-Pfalz” (handling code 837.035.09 (6540)) and by the competent authority “Bundesinstitut für Arzneimittel und Medizinprodukte” (06/03/2009; handling code 61-3910-4035002) were obtained before the study was started. The clinical trial was registered at ClinicalTrials.gov (identifier: NCT00878917) before the first patient was screened. Before inclusion, informed consent was obtained from every patient. The study was performed following the tenets of the Declaration of Helsinki. According to the sample size calculation, thirty-two patients were required.

### 2.1. Inclusion and Exclusion Criteria

The inclusion and exclusion criteria are given in Table 1.

If both the eyes qualified, the eye with the higher IOP was chosen for evaluation or if IOP was the same for both the eyes, the eye with the worse visual acuity was selected.

### 2.2. Study Treatments

The inclusion criteria were not in accordance with the indications for treatment mentioned in the German summary of medicinal product characteristics (“Fachinformation”) for Trusopt® Eye drops (TRUS-GPC-2006 11 08) due to the fact that Trusopt was used as monotherapy and not in combination with beta-blockers. In this study, we referred to the guidelines of the “European Glaucoma Society” [14]. The guidelines state that dorzolamide 2% eye drops are appropriate as the first-choice treatment for monotherapy of primary open-angle glaucoma.

#### 2.2.1. Test Product

Dorzolamide 2% eye drops solution containing dorzolamide 20 mg/ml as hydrochloride eye drops solution (Rompharm Company SRL, Romania) was used. The final formula for dorzolamide 2% eye drops solution is given in Table 2.

#### 2.2.2. Reference Therapy

Trusopt® eye drops solution (2%) containing dorzolamide 20 mg/ml as hydrochloride (Chibret Pharmazeutische GmbH, Germany) was used.

On each container, there was masked labeling; thus, the patient and the study personnel did not know which medicine the participant was taking. All study personnel and the investigators performing investigations and/or assessments during the trial days were masked to the study of medicine.

Each patient was randomized to one of two treatment sequences (*n* = 16 per group), either with the test (*T*) product for 4 weeks followed by the reference (*R*) product for 4 weeks or the reference product for 4 weeks followed by the test product for 4 weeks. Randomization was performed by staff of the trial center. The randomization list was kept in safe and confidential custody by persons who did not enroll or treat study patients. The randomization contained one randomization block of size 32 (16 patients per treatment sequence). No stratification was done. The clinical trial site received a set of sealed envelopes, one for each patient. Before the first treatment sequence was initiated, a washout was performed according to the washout scheme: miotics and oral/topical carbonic anhydrase inhibitors for 5 days, alpha and alpha/beta-agonists for 14 days; and beta-antagonists and prostaglandins for 28 days.

A one week washout was performed between both products. Patients received one drop three times a day (at 08.00, 15.00, and 22.00) for the duration of each treatment phase.

The primary efficacy endpoint was the difference in mean diurnal IOP from visit 1 to visit 2 and from visit 3 to visit 4 in the study eye (per-protocol population). Patients who discontinued study treatment because of lack of IOP-lowering efficacy had their last IOP observation carried forward in the per-protocol analysis. The safety analysis was based on an intent-to-treat dataset and included all patients who received study medication. The secondary efficacy parameter was the difference in diurnal IOPs at each individual time point from visit 1 to visit 2 and from visit 3 to visit 4. One eye per patient was considered in this trial.

Both the eyes were treated with study medication during the study if the other eye required treatment as well, but the second eye was not used as the study eye. Four visits as well as a screening visit were performed throughout the study. If the patient did not take any prior IOP-lowering medication, screening and baseline visits were performed on the same day. The washout phase before baseline and between the two treatment sequences (7 days) was kept as short as possible and according to the recommendations of the European Glaucoma Society: Terminology and Guidelines for Glaucoma [14]. This was a requirement by the ethics committee to minimize the risks for the study subjects.  Table 3 provides the trial schedule. At the inclusion visit, the mode of administration of the test product and reference therapy was explained to each patient in detail. A follow-up booklet, in which the patient should report any deviations from the stipulated frequency of application, any problems of local tolerability, any adverse reactions, and any comments related to the study, was handed to each patient. At visits 1 and 3, the first application of study medication was at 16 : 00 and was performed by study personnel. Fifteen minutes after the first application, the patient was asked about any side effects. At the last visit, all the investigational medicinal products (full, half-used, or empty) had to be returned to the investigator. At each visit, any change in the frequency of administration of the test product was recorded in the CRF. This evaluation was based on the data recorded in the follow-up booklet given to each patient. As an additional measure of compliance, the bottles were weighed before distribution to and after recollecting from the patients.

#### 2.2.3. The following Concomitant Treatments Were Not Permitted during the Trial

Use of any systemic medication would affect IOP with less than a 1-month stable dosing regimen before the screening visit (i.e., steroids) and use of any additional local treatment affects IOP. If the patient reported prohibited treatment, exclusion of the trial was discussed. Goldmann applanation tonometry was performed at 8 : 00, 12 : 00, and 16 : 00 (±1 h) at each visit. For this, both the eyes were anesthetized with a solution containing oxybuprocaine HCl 4 mg and fluorescein sodium (Thilorbin® eye drops, Alcon Pharma GmbH, Freiburg, Germany), and the Goldmann tonometer dial was set to 10 mmHg. After blindly adjusting the dial until the inner edge of the fluorescein mires touched slightly, the investigator then recorded the IOP value, reset the tonometer to 10 mmHg, and repeated the process. Right eyes were always measured before left eyes. The IOP values (rounded to the nearest whole number) at each time point were recorded as the mean of 2 measurements within 2 mmHg or the mean of 3 measurements if the first 2 measurements differed by ≥ 3 mmHg. The IOP(t) at single time points were reported as measured with one digit only. The averaged values IOPav = (IOP(8 : 00) + IOP(12 : 00) + IOP(16 : 00))/3 were reported with 2 digits. All IOP measurements were performed by 1 of 3 experienced investigators, and the same tonometer was used on each patient at all visits. The calibration of the tonometer was checked once a month. Safety was assessed by documentation of adverse events (AEs) and serious adverse events (SAEs).

### 2.3. Statistical Methods

An average reduction of the IOP from the period baseline of *δ*IOPav = 3.0 mmHg with an intrasubject variability of ±2 mmHg was assumed with dorzolamide treatment. A difference of 1.5 mmHg was considered as clinically not relevant and thus acceptable. Equivalence thus was accepted if the 90% confidence interval for the treatment difference *δ*IOPav test−*δ*IOPav reference was included by an acceptance range of ±1.5 mmHg, which additionally is in agreement with equivalence for IOP-lowering drugs necessary for the FDA [25]. Thirty-two patients were required to demonstrate equivalence with a power of 80% if the true difference between the formulations was zero.

The prespecified analysis was an ANOVA with treatment, sequence, study period, and patient within the sequence as effects. Model assumptions were checked by testing the residuals for nonnormality. Sequence and period effects were also investigated. For the difference, *δ*IOPav test−*δ*IOPav reference parametric point estimators for the treatment differences and 90% confidence intervals were calculated. However, the analysis of the study data revealed a much higher intrasubject variability than anticipated, especially for the baseline IOP between the study periods. Therefore, a post hoc ANCOVA including the baseline IOP was performed in addition. The analysis was repeated also for the single time points 8 : 00, 12 : 00, and 16 : 00.

## 3. Results

**Figure 1:**
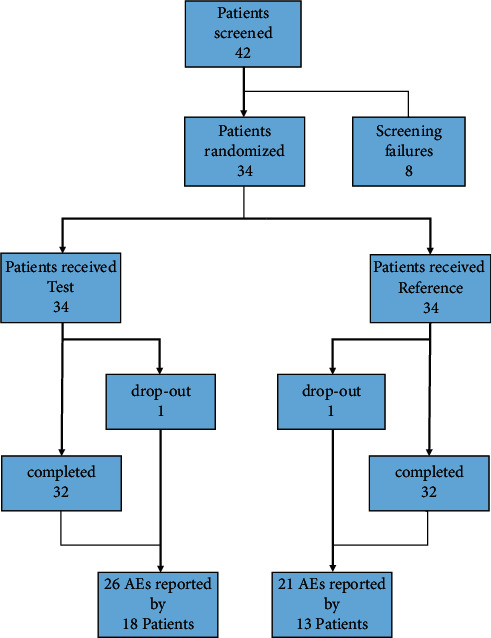
Study overview.

Forty-two patients were screened in total, and 34 patients were enrolled and randomized for the trial (20 female and 14 male). The clinical part of the study was performed between 15 May 2009 (first screening examination) and 29 January 2010 (last patient's last visit). Twenty female and 14 male patients were analyzed in the safety population (Figure 1). The mean age of the safety population was 65.1 (range 47–83) years for females and 67.6 (range 59–74) years for male patients. All patients were Caucasians. Two patients dropped out during the study; therefore, the per-protocol population included 32 patients and the safety population included all 34 patients, as shown in Figure 1.

Forty-two patients were screened, of which 8 patients were not included in the study due to screening failure. Patients were randomized to either the first test and second reference product or first reference and second test product. 2 patients dropped out during the trial (1 in each sequence). 32 patients completed the trial. 26 adverse events were reported when test product was applied, and 21 adverse events were reported during reference product usage.

The primary efficacy parameter was the difference in mean diurnal IOP from visit 1 (baseline) to visit 2 and from visit 3 (baseline) to visit 4. The ANOVA and ANCOVA residuals were normally distributed, and there was no significant (*p* > 0.05) period and no sequence effect suggesting a carry-over effect. Using the ANOVA model anticipated in the statistical analysis plan, the 90% confidence interval for the primary endpoint the *δ*IOPav difference (−0.53 mmHg (−1.58 mmHg–0.52 mmHg)) appeared to miss the anticipated acceptance range for equivalence of ±1.5 mmHg. According to the self-report in the patient booklet, all 32 patients were >80% compliant. After the closure of the database and analysis of the primary endpoint, the compliance was rechecked. It became obvious that the weight of the bottle containers and the self-reported entries in the patient booklets were not conformed. Two patients reported more than 80% compliance, but according to the weight of their bottles, we concluded that they could not have used more than 50% of their eye drops solution. The two subjects who should have been excluded due to compliance issues had a remarkable effect on the analysis of equivalence (see the extent of exposure). An analysis without these two patients revealed equivalence of both tested formulations.

The premedication baseline in each period is a significant (*p* value<0.01) source of variation in the ANCOVA model using the baseline IOP as a covariate. In the ANCOVA model, the intrasubject variability could be reduced to ±2.1 mmHg from ±2.48 mmHg of the model without covariate and the differences between the treatments were halved to −0.27 mmHg. Under the ANCOVA model, the 90% confidence interval for the average change of the IOP −0.27 mmHg (−1.17 mmHg–0.64 mmHg) is included by the acceptance range −1.5 mmHg to +1.5 mmHg as stipulated in the study protocol. The same is true for the changes of the IOP at discrete time points, which all meet the acceptance range. There was no statistically significant difference in IOP at each time point at 8 : 00, 12 : 00, and 16 : 00 between the two drugs. Even the 95% confidence interval (−1.36 mmHg–0.82 mmHg) was in the acceptance range.

Similar relationships for the average IOP change (*δ*IOPav) could be demonstrated also for the changes at discrete time points. The respective regression coefficients were 0.8537, 0.7071, and 0.830 for *δ*IOP8:00, *δ*IOP12 : 00, and *δ*IOP16 : 00, respectively. The summary statistics for the average IOP in the study eye are given in Table 4.

All 34 patients treated with study medication were included in the safety evaluation. One drop-out patient only received the test product and the other only the reference therapy. Total individual drug exposure of the 32 patients who were dosed according to protocol differed between 2.37 g and 11.7 g of reference therapy and 2.61–11.59 g of test product during the study. 4 patients treated only one eye, which explains the lower consumption of medication in these patients. Only after the closure of the database and after unmasking, it became apparent that in 2 patients, both the eyes had been treated, which had been wrongly interpreted by the data management. The patients therefore could have used only about 50%–60% of the expected amount of study medication. It appeared illegitimate to revise the decision made in the data review meeting and to include these patients into the per-protocol population, after unmasking. Adverse events (AEs) during this study were evaluated as a secondary parameter. A detailed list of the AEs can be found in the supplementary Table 1.

Twenty-six of the 34 patients treated reported altogether 54 AEs: 26 AEs were reported by 18 patients after the administration of the test product and 21 AEs were reported by 13 patients after reference therapy, respectively. The reported AEs were eye disorders (12 patients), gastrointestinal disorders (5 patients), nervous system disorders (5 patients), injury, poisoning and procedural complications (4 patients), musculoskeletal and connective tissue disorders (4 patients), general disorders and administration site conditions (3 patients), and respiratory, thoracic, and mediastinal disorders (3 patients). Ear and labyrinth disorders, infections and infestations, investigations, psychiatric disorders, and skin and subcutaneous tissue disorders occurred only in one patient consecutively. Thirty-seven events were resolved completely. In 10 AEs, the outcome improved; in 4 cases, the outcome was unchanged, and in 2 cases, it is unknown. Serious adverse events were found in three patients: one patient with synovitis of the left shoulder, one patient with an accident with a chain saw involving a laceration of the leg, and one patient with epistaxis (led to the withdrawal of consent and drop-out of the patient).

The intensity of the AEs was rated as severe in 3 cases, moderate in 19 cases, and mild in all other 31 cases. 12 AEs were suspected as related to the study drug, 2 AEs were classified as probably related, 16 AEs were suspected as possibly related, 8 AEs were classified as unlikely related, and 16 AEs were classified as not related to the study drug. There was no clinically relevant difference observed in frequency or severity of the AEs between test and reference treatment. There were three SAEs, and they were not related to the study medication.

Best-corrected visual acuity stayed stable throughout the study in all patients. The major findings in the slit-lamp examination were lid edema in 4 patients, lid erythema in 3 patients, conjunctival erythema in 29 patients, conjunctival edema in 6 patients, and epithelial defects in 11 patients. At visit 2 and visit 4, patients had to answer an ocular symptoms questionnaire. The answers showed no clinically relevant effects concerning the safety or differences between the two tested treatments. The tested product received registration in 11 European countries in a decentralized procedure (Table 5).

## 4. Discussion

This monocentric randomized double-masked active-controlled clinical trial with a crossover design was performed to demonstrate the IOP-lowering efficacy of generic dorzolamide 2% eye drops solution applied three times a day (t.i.d) (containing dorzolamide 20 mg/ml as hydrochloride, manufactured by Rompharm Company SRL, Romania) (test product) is equivalent to that of Trusopt® eye drops solution (2%) applied t.i.d. (containing dorzolamide 20 mg/ml as hydrochloride, manufactured by Chibret Pharmazeutische GmbH, Germany) in patients with open-angle glaucoma or ocular hypertension.

The tested eye drop solution is designed as a generic drug for use on the European market. The European Medicine Agency (EMA) defines a generic drug as being of the same active substance as the already authorized reference medicine; however, inactive ingredients can differ. As studies showing the safety and efficacy of the reference product have already been performed, merely the quality of the medicine and the equivalent bioavailability had to be demonstrated (EMA/393905/2006 Rev. 2). As the tested solution shows a different viscosity to the reference product, a clinical trial showing equivalence of IOP-lowering properties was necessary. The standard study design for a generic medicine for the EMA is a randomized, two-period, two-sequence, single-dose crossover design, as performed here in this trial [26]. Equivalence was not reached in this trial. *δ*IOPav difference (−0.53 mmHg (−1.58 mmHg–0.52 mmHg)) just failed to meet the equivalence bounds. However, for the following reasons, the competent authorities still accepted the data of this trial for registration of generic dorzolamide.

The study was conducted in adherence to the study protocol in compliance with GCP (Good Clinical Practice). In the unblinded data review phone conference, the study team agreed that there were no major protocol deviations likely to impair the evaluation of the study's endpoints for the 32 patients that finished both study periods according to the protocol. Only after the closure of the database and unmasking the data, it became apparent that severe compliance issues had occurred in two patients. These two patients had treated both the eyes instead of one eye as misleadingly reported in the case report forms. The weight of the corresponding study medication containers revealed maximum compliance of 50–60%, which was in contrast to reported compliance of more than 80% in the patient diaries. This led to the conclusion that these two patients should have been excluded from the analysis due to insufficient compliance.

Insufficient adherence to medication is known to be a general problem in glaucoma patients and can lead to increased visual field progression [27]. A wide range of rates of compliance varying from 5% to 80% has been reported [28]. This is not only challenging for clinical trials but also in an everyday clinical setting, evaluating glaucoma progression despite good IOP values challenging for the treating physician [29]. A possible white-coat phenomenon leading to regular treatment usage approximately 5 days before seeing a specialist decreases the likelihood of detecting nonadherent patients [30]. A recent study analyzing reasons for nonadherence in long-term glaucoma patients demonstrated complete medication adherence of just about 41% of the patients, whereas the majority of the patients presented with moderate compliance (54%) [31]. The necessity of having to take a pill or apply the drop t.i.d. can also reduce patient compliance [32] and as being a chronic disease, causing no pain, adherence especially in not sight-threatening glaucoma is known to be low [33]. Experiencing side effects such as those reported within this trial can also limit patient adherence [33]. No clinically relevant differences in incidence or pattern of AEs were found between the two treatments investigated, although slightly more incidences were reported after using the test product. This could be due to the more gelatinous texture of the test solution, which possibly can lead to an extended exposure time on the ocular surface and might cause more side effects. Altogether, 53 adverse events (AEs) were observed during the study; in 30 of these AEs, the relationship was assessed as suspected (related, probably related, or possibly related) to the study drugs: 15 AEs occurred after administration of test medication and 15 AEs were documented while patients were on reference treatment. The most frequent AEs were eye disorders, followed by dysgeusia, dry mouth, and other common side effects of dorzolamide that were already described in the German summary of medicinal product characteristics. As performed in this trial, self-reported adherence can result in overestimation of patient compliance [29]; therefore, additional eye drop weighting was performed in this study. Although these additional measurements may not be valid to detect moderately noncompliant patients, we were able to identify participants with very low compliance. Weight variations were possible as patients were encouraged to try and instill another drop in case the first attempt missed the eye, until making sure the drop reached the eye. Elimination of the two severely noncompliant patients from the analysis was able to reveal the actual equivalence of the generic test product to the reference product and lead to acceptance of the generic dorzolamide for registration. Considering the costs of clinical trials and the consequences for drug companies, this study shows that special focus should not only lie on the trial design but also on trial adherence measurements.

A baseline IOP between 18 and 32 mmHg was required to randomize the patient in one of the three measurements in the study eye (at 8 : 00, 12 : 00, or 16 : 00 h). However, of all the patients demonstrating at least one measurement within the limits given as inclusion criteria, 25% demonstrated a low averaged diurnal baseline IOP of ≤18 mmHg. This inclusion criterion retrospectively appears to be too liberal and could partly be due to the short washout period. The motivation to perform a pharmacodynamic study in patients instead of healthy volunteers under highly standardized conditions is the assumption that the desired pharmacodynamic effect in patients with a higher IOP is more pronounced than in healthy volunteers with an IOP in the normal range. A pronounced IOP depressing effect seems less likely and may not be expected if baseline IOP is already low.

When comparing the IOP deceasing effects of the test vs. the reference product, a high variability was found. Introducing the premedication baseline IOP (at visits 1 and 3, respectively) as a covariate into the ANCOVA model led to reduction of the intrasubject variability from ±2.48 mmHg without covariate to ±2.11 mmHg. The consideration of the covariate in the model reduced not only the variability but also adjusted the Apriori differences of the baseline IOP between the two treatment groups (T: 20.01 mmHg, R: 20.35 mmHg). Under the ANCOVA model, the 90% confidence interval for the average change of the IOP became −0.27 mmHg (−1.17 mmHg–0.64 mmHg). This is within the acceptance range of −1.5 mmHg to +1.5 mmHg stipulated in the study protocol. The ANCOVA model substantially improved the precision of the analysis. These results suggest equivalence for the tested formulations with respect to the extent of the IOP decrease.

Another reason for the higher variability of the IOP decreasing effects could have been the fact that different investigators had determined the IOP in a patient at different visits or even at the same visit but at different time points. The intra and intersubject variabilities in Goldmann applanation tonometry are well known [34] and possibly could play a role, although the examiners all were trained and experienced. Cycloplegic drugs, in this study for dilated fundus examination at the screening visit and visit 4 as a safety parameter, can also cause a significant rise in IOP in patients with open-angle glaucoma [35].

## 5. Conclusions

This study showed the equivalence of the tested generic dorzolamide (dorzolamide 2% eye drops solution, containing dorzolamide 20 mg/ml as hydrochloride, manufactured by Rompharm Company SRL, Romania) to the reference product Trusopt® eye drops solution (2%) containing dorzolamide 20 mg/ml as hydrochloride (Chibret Pharmazeutische GmbH, Germany). Therefore, the tested product received registration in 11 European countries in a decentralized procedure. In addition, this clinical trial shows that special precautions should be taken to determine patient adherence to the study medication. This could not only prevent potential drugs from reaching the market but is also relevant for companies investing in expensive clinical trials.

## Figures and Tables

**Table 1:** Inclusion and exclusion criteria.

Inclusion criteria	
Age >18 years	Male/female, all races
Primary open-angle glaucoma, pseudoexfoliation glaucoma, pigment dispersion glaucoma, and ocular hypertension	In one or both the eyes
Baseline IOP after washout (study eye)	IOP between 18 and 32 mmHg in one of the 3 measurements (in the eyes not included in the study (fellow eye); IOP must have been controllable on no pharmacologic treatment or on the study medicine only)
Best-corrected distance visual acuity	20/200 (Snellen equivalent) or better in the study eye

Exclusion criteria	
Patients presenting one of the following criteria were excluded from study enrollment.	
Chronic or recurrent inflammatory eye disease	
Ocular trauma within the past six months	
Current ocular infection, i.e., conjunctivitis or keratitis	
Any abnormality preventing reliable applanation tonometry	
Intraocular surgery or laser treatment within the past three months	
Inability to discontinue contact lens wear during the study	
Use of any systemic medication that would affect IOP with less than a 1-month stable dosing regimen before the screening visit	
Patient allergic to sulfonamides	
Severe renal dysfunction or hyperchloraemic acidosis	

**Table 2:** Final formula for dorzolamide 2% eye drops solution.

No.	Material	Amount	Function
1	Dorzolamide hydrochloride (corresponding to dorzolamide)	22.30 mg (20.0 mg)	Active substance
2	Hydroxyethyl cellulose	1.00 mg	Viscosity-increasing agent
3	Citric acid monohydrate	4.00 mg	Buffering agent
4	Sodium hydroxide	2.26 mg	Buffering agent
5	Mannitol	20.00 mg	Tonicity agent
6	Benzalkonium chloride	0.075 mg	Antimicrobial preservative
7	Purified water	1.0 mL	Vehicle

For the innovator formula, no details are published and available.

**Table 3:** Trial schedule.

Visit action	Screening	Visit 1	Visit 2	Visit 3	Visit 4
Trial day	Day −28 to 1	Day 1	Day 28 ± 5	Day 35−1/+3	Day 63 ± 5
Demographics (sex, age)	x				
Patient information and informed consent	X				
Previous and concomitant diseases	X				
Previous and concomitant treatments	X				
Inclusion/exclusion criteria	X				
Vital signs (blood pressure, pulse)	X		X		X
Best-corrected visual acuity	X	X	X	X	X
Pregnancy test (if woman of childbearing potential)	X				
Laboratory tests	X		X		X
IOP (8.00, 12.00, and 16.00 ± 1 h)	X	X	X	X	X
Application of study medication by study personnel (16 : 00 ± 1 h, after IOP measurement)		X		X	
Randomization		X			
Slit-lamp examination	X	X	X	X	X
Dilated fundus examination	X				X
Dispensation of study medication		X		X	
Symptom survey			X		X
Changes in medical health or concomitant medication		X	X	X	X
Adverse events		X	X	X	X
End of trial (final visit)					X

**Table 4:** Summary statistics for the average IOP in the study eye.

Variable	Statistics	*T*	*R*	T-R
Baseline IOP_av_ (mmHg)	*N*	32	32	32
	Mean	20.01	20.35	−0.34
	Standard deviation	3.04	3.11	2.51
	Min	14.67	16.00	−6.33
	Median	19.67	19.58	0.00
	Max	27.33	27.50	5.33

IOP_av_ (mmHg)	Mean	17.83	17.65	0.19
	Standard deviation	3.55	2.82	3.03
	Min	13.67	13.00	−5.00
	Median	17.33	17.83	0.58
	Max	28.00	24.00	9.50

*δ*IOP_av_^*∗*^ (mmHg)	Mean	2.18	2.70	−0.52
	Standard deviation	2.36	2.76	3.45
	Min	−2.67	−2.67	−8.83
	Median	2.33	3.00	−0.08
	Max	6.67	8.50	4.83

^*∗*^The signs of the baseline-corrected effects are positive (the larger the desired effect, the larger the value). The negative T-R *δ*IOPav indicates a higher average IOP reduction of 0.52 mmHG for the reference/original product. *T*, test product; *R*, reference product.

**Table 5:** Countries where generic dorzolamide was registered and their trade names.

Country	Trade name
Hungary, Latvia, Poland, Slovakia	Latalux
Germany, Austria, Denmark, Finland, Norway, Sweden	Dorzolamide Tiefenbacher
France	Dorzolamide Alfred E. Tiefenbacher
Germany, Denmark, Norway, Sweden	Dorat 20 mg/ml
Germany, France	Zolasopt 20 mg/ml

## Data Availability

The datasets during and/or analyzed during the current study are available from the corresponding author upon request.

## Supplementary Materials

Supplementary Materials Supplementary Table 1 provides the number and percent of patients reporting adverse events, by system organ class, preferred term, and treatment.
