# Supplementary material for: A Randomized, Double-Masked, Active-Controlled, Crossover Phase III Equivalence Study of Generic Dorzolamide 2% versus Innovator Trusopt® Eye Drop Solution in Subjects with Open-Angle Glaucoma or Ocular Hypertension
Source: J Ophthalmol. 2022 Jul 20;2022:5249922. doi: 10.1155/2022/5249922 (PMC9329021; doi:10.1155/2022/5249922)
Supplement: Supplementary Materials — Supplementary Table 1 provides the number and percent of patients reporting adverse events, by system organ class, preferred term, and treatment. [file 5249922.f1.docx]

**Table suppl. 1: Number and percent of patients reporting adverse events**[**^1)^,**](#_bookmark88) **by system organ class, preferred term and treatment**[**^2)^**](#_bookmark89)

| **System Organ Class/ Preferred Term** | **Treatment**  **Reference (N=33)** | **Treatment**  **Test (N=33)** | **Total (N=34)** |
| --- | --- | --- | --- |
| Total number (%) of patients with AE | 13 (39%) | 18 (55%) | 26 (76%) |
| Ear and labyrinth disorders | 0 (0%) | 1 (3%) | 1 (3%) |
| Vertigo | 0 (0%) | 1 (3%) | 1 (3%) |
| Eye disorders | 5 (15%) | 8 (24%) | 12 (35%) |
| Blepharitis | 3 (9%) | 1 (3%) | 3 (9%) |
| Eye haemorrhage | 1 (3%) | 1 (3%) | 2 (6%) |
| Abnormal sensation in eye | 0 (0%) | 1 (3%) | 1 (3%) |
| Asthenopia | 1 (3%) | 0 (0%) | 1 (3%) |
| Conjunctival haemorrhage | 0 (0%) | 1 (3%) | 1 (3%) |
| Corneal epithelium defect | 0 (0%) | 1 (3%) | 1 (3%) |
| Dry eye | 0 (0%) | 1 (3%) | 1 (3%) |
| Eye allergy | 0 (0%) | 1 (3%) | 1 (3%) |
| Myodesopsia | 1 (3%) | 0 (0%) | 1 (3%) |
| Photophobia | 0 (0%) | 1 (3%) | 1 (3%) |
| Visual acuity reduced transiently | 0 (0%) | 1 (3%) | 1 (3%) |
| Gastrointestinal disorders | 4 (12%) | 1 (3%) | 5 (15%) |
| Dry mouth | 2 (6%) | 0 (0%) | 2 (6%) |
| Nausea | 1 (3%) | 1 (3%) | 2 (6%) |
| Diarrhoea | 1 (3%) | 0 (0%) | 1 (3%) |
| Toothache | 1 (3%) | 0 (0%) | 1 (3%) |
| General disorders and administration site | 1 (3%) | 2 (6%) | 3 (9%) |
| conditions |  |  |  |
| Application site pain | 0 (0%) | 1 (3%) | 1 (3%) |
| Drug intolerance | 0 (0%) | 1 (3%) | 1 (3%) |
| Fatigue | 1 (3%) | 0 (0%) | 1 (3%) |
| Infections and infestations | 1 (3%) | 0 (0%) | 1 (3%) |
| Otitis media | 1 (3%) | 0 (0%) | 1 (3%) |
| Injury, poisoning and procedural complica- | 2 (6%) | 2 (6%) | 4 (12%) |
| tions |  |  |  |
| Arthropod sting | 1 (3%) | 0 (0%) | 1 (3%) |
| Muscle strain | 0 (0%) | 1 (3%) | 1 (3%) |
| Skin laceration | 1 (3%) | 0 (0%) | 1 (3%) |
| Thermal burn | 0 (0%) | 1 (3%) | 1 (3%) |
| Investigations | 0 (0%) | 1 (3%) | 1 (3%) |
| Blood amylase increased | 0 (0%) | 1 (3%) | 1 (3%) |
| Musculoskeletal and connective tissue dis- | 1 (3%) | 3 (9%) | 4 (12%) |
| orders |  |  |  |

1) occurred after 1st administration

2) N = Number of subjects treated

**Table suppl. 1: Number and percent of patients reporting adverse events^1)^, by system organ class, preferred term and treatment^2)^**

| **System Organ Class/ Preferred Term** | **Treatment Reference**  **(N=33)** | | | **Treatment Test**  **(N=33)** | | | **Total (N=34)** |
| --- | --- | --- | --- | --- | --- | --- | --- |
| Back pain | 0 | ( | 0%) | 1 | ( | 3%) | 1 (3%) |
| Musculoskeletal pain | 0 | ( | 0%) | 1 | ( | 3%) | 1 (3%) |
| Myalgia | 1 | ( | 3%) | 0 | ( | 0%) | 1 (3%) |
| Synovitis | 0 | ( | 0%) | 1 | ( | 3%) | 1 (3%) |
| Nervous system disorders | 2 | ( | 6%) | 3 | ( | 9%) | 5 (15%) |
| Dysgeusia | 1 | ( | 3%) | 2 | ( | 6%) | 3 (9%) |
| Headache | 1 | ( | 3%) | 1 | ( | 3%) | 2 (6%) |
| Psychiatric disorders | 0 | ( | 0%) | 1 | ( | 3%) | 1 (3%) |
| Insomnia | 0 | ( | 0%) | 1 | ( | 3%) | 1 (3%) |
| Respiratory, thoracic and mediastinal disor- ders | 2 | ( | 6%) | 1 | ( | 3%) | 3 (9%) |
| Epistaxis | 1 | ( | 3%) | 0 | ( | 0%) | 1 (3%) |
| Nasal dryness | 1 | ( | 3%) | 0 | ( | 0%) | 1 (3%) |
| Oropharyngeal pain | 0 | ( | 0%) | 1 | ( | 3%) | 1 (3%) |
| Skin and subcutaneous tissue disorders | 0 | ( | 0%) | 1 | ( | 3%) | 1 (3%) |
| Hyperhidrosis | 0 | ( | 0%) | 1 | ( | 3%) | 1 (3%) |
